# Monitoring spindle orientation: Spindle position checkpoint in charge

**DOI:** 10.1186/1747-1028-5-28

**Published:** 2010-12-11

**Authors:** Ayse K Caydasi, Bashar Ibrahim, Gislene Pereira

**Affiliations:** 1 German Cancer Research Centre, DKFZ-ZMBH Alliance, Molecular Biology of Centrosomes and Cilia, Im Neuenheimer Feld 581, 69120 Heidelberg, Germany

## Abstract

Every cell division in budding yeast is inherently asymmetric and counts on the correct positioning of the mitotic spindle along the mother-daughter polarity axis for faithful chromosome segregation. A surveillance mechanism named the spindle position checkpoint (SPOC), monitors the orientation of the mitotic spindle and prevents cells from exiting mitosis when the spindle fails to align along the mother-daughter axis. SPOC is essential for maintenance of ploidy in budding yeast and similar mechanisms might exist in higher eukaryotes to ensure faithful asymmetric cell division. Here, we review the current model of SPOC activation and highlight the importance of protein localization and phosphorylation for SPOC function.

## Introduction

**Figure 1:**
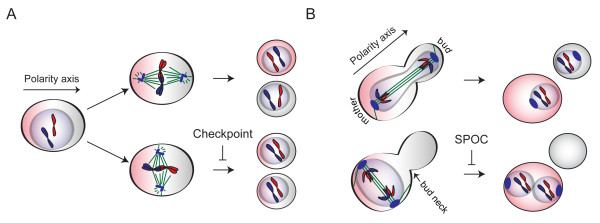
**Impact of spindle orientation on asymmetric cell division**. Asymmetric cell division is depicted in a hypothetical polarized cell (A) and in budding yeast (B). Only two chromosomes are shown for simplicity. In the upper panels, spindle aligns along the polarity axis and asymmetric cell division ends successfully giving rise to two different cells which carry different cell fate determinants depicted in different colors. In the lower panels, spindle aligns perpendicular to the polarity axis which results in failure of the asymmetric cell division in A and aneuploidy in B. Note that, the site of cell division in budding yeast is determined at G1/S which is before spindle assembly and entry into mitosis. Hence, if budding yeast divides despite the failure of spindle alignment along the polarity axis, inheritance of the cell fate determinants is not affected but aneuploidy occurs.

Positioning of the mitotic spindle with respect to the polarity axis becomes important during asymmetric cell division. In many polarized cells that place the cleavage furrow in relation to the position of the mitotic spindle, orientation of the spindle determines the fate of the two daughter cells without affecting the accuracy of chromosome segregation (Figure 1A). However in *S. cerevisiae*, spindle alignment along the polarity axis is particularly crucial for fidelity of chromosome segregation. This is mainly because of the physical constrains that arise from the establishment of the site of cell division (bud neck) before entry into mitosis (Figure 1B).

In budding yeast, a faithful mitosis requires positioning of the mitotic spindle along the mother-bud axis to ensure that the expanding anaphase spindle leaves one set of chromosomes in the mother cell while the second set is dragged through the bud neck into the daughter cell (Figure 1B). Misalignment of the mitotic spindle eventually leads to aneuploidy. Therefore, yeast cells have developed several mechanisms to provide correct spindle alignment. Firstly, spindle positioning in budding yeast is achieved by two functionally redundant microtubule-associated pathways, one containing the Kar9 protein and the other containing the minus-end-directed motor protein dynein [1-8]. Impairment of either pathway brings about spindle misorientation in nearly 10-20% of the cells, while impairment of both is lethal [1,7]. Secondly, to prevent cells exiting mitosis with misaligned spindles, budding yeast have evolved a surveillance mechanism known as the spindle position checkpoint (SPOC) [9-12]. Mutants affecting the function of either the *KAR9 *or *DYN1 *pathway genes frequently misalign their spindles and rely on SPOC for survival [10]. SPOC delays the exit from mitosis by inhibiting the mitotic exit network (MEN) in response to spindle orientation defects. SPOC inhibition of MEN involves phosphorylation events and alterations in the localization of proteins.

This review aims to assemble the recent advances in the SPOC field into a model. Starting from mitotic exit in budding yeast, we will focus on how SPOC inhibits MEN and how SPOC components are regulated.

### Exit from mitosis in budding yeast

**Figure 2:**
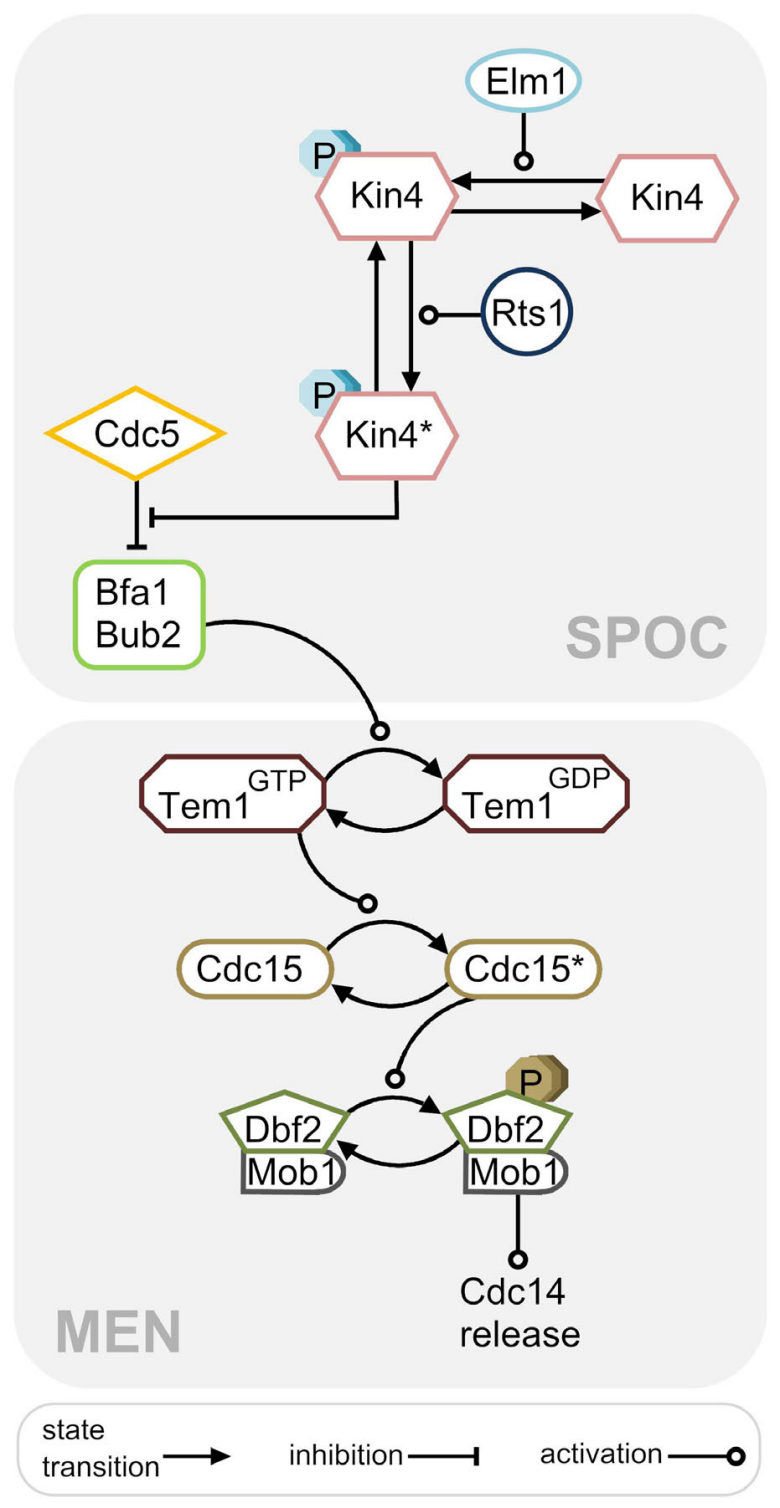
**MEN and SPOC**. Schematic representation of mitotic exit network and spindle position checkpoint. See the text for details on both SPOC and MEN pathways. Asterisks indicate the SPB localized forms of the corresponding protein.

Mitosis in budding yeast is driven by the activity of the sole cyclin dependent kinase (Cdk) Cdc28 in complex with mitotic cyclins (Clb1-4) [13-15]. Consequently, mitotic exit requires inactivation of the mitotic cyclin-Cdk complex and reversal of the Cdk dependent phosphorylation of several Cdk substrates. In budding yeast, a conserved dual specificity protein phosphatase called Cdc14 is capable of performing both these functions [16-18]. Activation of Cdc14 occurs in two steps, each of which involves the alteration of Cdc14 localization and hence the availability of Cdc14 for its substrates. From G1 until anaphase Cdc14 is kept inactive and sequestered in the nucleolus in association with its inhibitor Net1 [19-21]. The first step of activation takes place in early anaphase by partial release of Cdc14 from the nucleolus into the nucleoplasm and to some extend into the cytoplasm. This process is driven by the cdc-fourteen early anaphase release (FEAR) network which promotes Cdk dependent phosphorylation of Net1 [22-27]. FEAR dependent activation of Cdc14 is not essential for mitotic exit but it is crucial for the anaphase related tasks such as positioning of the anaphase nucleus, stabilization of the anaphase spindle, spindle midzone assembly and segregation of ribosomal DNA [28-35]. Full release of Cdc14 from nucleolus into the cytoplasm requires another step which is governed by the mitotic exit network (MEN) [19] (Figure 2). Unlike FEAR, MEN is essential for mitotic exit [36].

MEN was first proposed as an essential pathway for mitotic exit, by the analysis of temperature sensitive mutants that arrest in late anaphase with high mitotic cyclin levels [36]. Since then, it has been well established that the function of MEN in mitotic exit is to promote the full release of Cdc14 out of the nucleolus and retention of Cdc14 in the cytoplasm. MEN has also been reported to be important in regulation of cytokinesis [37-39]. However, our main focus in this section will be regulation of mitotic exit by the MEN.

MEN is a signal transduction pathway driven by the Ras-like GTPase Tem1 (Figure 2). GTP-bound Tem1 binds to the downstream kinase Cdc15 at the spindle pole body (SPB, centrosome equivalent in yeast) [40,41]. This binding allows Cdc15 to activate the Dbf2-Mob1 kinase complex through phosphorylation of the Dbf2 kinase subunit [42-44]. Activated Dbf2-Mob1 translocates to the nucleus, promotes dissociation of Cdc14 from Net1 by a yet unknown mechanism and phosphorylates Cdc14 hindering its nuclear localization signal [45,46]. Hence Cdc14 liberated from the nucleolus cannot return to the nucleus once phosphorylated by Dbf2-Mob1. Cdc14 in the cytoplasm is now free to dephosphorylate its targets to promote mitotic exit. Key Cdc14 substrates include the mitotic Cdk inhibitor Sic1, the transcription factor Swi5 and the anaphase promoting complex (APC) activator Cdh1 [16,47].

Tem1 is supposedly active in the GTP bound state by similarity to its fission yeast homologue Spg1 [48]. The two-component GTPase activating protein (GAP) complex Bfa1-Bub2 inhibits Tem1 activity by promoting GTP hydrolysis [49]. The putative guanine nucleotide exchange factor (GEF), Lte1, presumably promotes Tem1 activation in the daughter cell compartment, although the molecular basis of this activation is still unclear. Lte1 has a GEF domain homologous to that of Cdc25 [50]. In addition, *lte1*Δ cells fail to exit mitosis at low temperatures. This mitotic exit defect is suppressed by a high copy number of *TEM1 *[41]. These observations led to the hypothesis that Lte1 is the GEF for Tem1. However, the fact that Lte1 is only essential at growth temperatures below 20°C suggests that Tem1 does not need a GEF at physiological temperatures. This could be explained by the high intrinsic GDP-to-GTP exchange activity of Tem1 [49]. Furthermore, the GEF domain of Lte1 is dispensable for mitotic exit activation at low temperatures, which also questions the GEF activity of Lte1 for Tem1 [51]. In fact, no Lte1 GEF activity was detected for Tem1 *in vitro *[52]. Instead, Lte1 appears to assist mitotic exit by an unknown mechanism, most likely via regulation of Bfa1 or Kin4 [52,53].

How is MEN activated? Polarity factors such as Rho-like GTPase Cdc42 and its effectors Cla4, Ste20, Gic1 and Gic2 promote mitotic exit mainly through targeting of Lte1 to the bud cortex and by interfering with Bfa1-Bub2 GAP function [54-57]. In addition, Cdc14 released via FEAR contributes to the MEN activity by Cdc15 and Dbf2-Mob1 activation through dephosphorylation of Cdk phosphorylated Cdc15 and Mob1 respectively and by promoting Bfa1-Bub2 inactivation through an unknown mechanism [58-60].

On the other hand, Cdc14 released via MEN eventually inactivates the MEN forming a negative feedback loop. Firstly, Cdc14 activates APC^Cdh1 ^which in turn promotes Cdc5 degradation [61]. Secondly, once released by the MEN, Cdc14 dephosphorylates Bfa1 promoting its re-activation [58]. In addition, fully activated Cdc14 triggers dissociation of Lte1 from the bud cortex through dephosphorylation, which leads to Lte1 inactivation [56,57]. Furthermore, Cdc14 induces transcription of the daughter-specific protein Amn1 which directly binds to Tem1 and prevents its interaction with Cdc15 [62]. This is achieved by activation of the transcription factors Swi5 and Ace2 through their dephosphorylation by Cdc14. Finally, Cdc14 returns into the nucleolus after it has dephosphorylated its targets, allowing the start of a new cell cycle [63].

### Regulation of MEN by SPOC

The *S. cerevisiae BUB2 *(budding uninhibited by benomyl) gene was originally identified in a genetic screen for mitotic checkpoint related genes required to delay cell cycle progression in response to microtubule defects (induced by microtubule depolymerizing drugs, benomyl and nocodazole) [64]. At that time, Bub2 was thought to be a part of the spindle assembly checkpoint (SAC; checkpoint that prevents metaphase to anaphase transition until all chromosomes accomplish bipolar attachment to the spindle microtubules) [64-67]. *BFA1 *(byr-four-alike-1) was discovered by homology to the fission yeast's Byr4 which together with Cdc16 (homologue of Bub2 in *S. pombe*) is involved in inhibition of the septation initiation network (SIN, homolog of MEN in *S. pombe*; pathway essential for cytokinesis and its coordination with mitosis) [68-70]. In subsequent studies it became evident that Bfa1 and Bub2 belonged to a different branch of mitotic checkpoint than the SAC [66,71-74]. It then became established that the Bfa1-Bub2 GAP complex constitutes a checkpoint that delays mitotic exit when the anaphase spindle fails to align in the mother-bud direction. This mechanism is now defined as the spindle position checkpoint (SPOC) [10,11] (Figure 2).

Bfa1 and Bub2 constitute a two-component GAP complex that activates GTP hydrolysis of Tem1. By doing so, it reduces the active form of Tem1 and inhibits mitotic exit. Despite the presence of a GAP homology domain (TBC), Bub2 alone does not affect the hydrolysis and dissociation of GTP from Tem1. However, Bub2 in the presence of Bfa1 increases the GTP dissociation and hydrolysis rate of Tem1 *in vitro *[49].

Bub2 and Bfa1 physically interact with each other and with Tem1 [10,75]. Bfa1 protein levels seem to be important for cell survival because overexpression of Bfa1 arrests cells in anaphase [74]. Also, the transcription and the protein levels of Bfa1 and Bub2 are stable during the cell cycle [75-77]. In contrast, the phosphorylation status of Bfa1 changes in a cell cycle dependent manner. During anaphase, Bfa1 is hyperphosphorylated by the polo like kinase Cdc5 and this form of Bfa1 cannot bind to Tem1 [77]. Consequently, when Bfa1 is phosphorylated by Cdc5, Bfa1-Bub2 GAP activity for Tem1 is inhibited *in vitro *[78]. Cdc5 thus phosphorylates Bfa1 in an inhibitory manner, favoring mitotic exit. Bub2 and Tem1 also undergo cell cycle dependent phosphorylation although the functional significance of this regulation is largely unclear [79,80].

Another kinase involved in Bfa1-Bub2 regulation is the Kin4 kinase. *KIN4 *was first identified as a genetic interactor of *KAR9 *in a large scale synthetic genetic array (SGA) analysis where the deletion of *KIN4 *decreased the survival of *kar9*Δ cells [81]. Following this, Kin4 was established as an essential component of the SPOC [82,83]. As such, the phenotype of *kin4*Δ is very similar to *bfa1*Δ or *bub2*Δ cells: all mutant cell types fail to arrest in response to spindle misalignment. Like Bfa1, high levels of Kin4 cause an anaphase arrest due to inactivation of the MEN that can be reverted by deletion of *BUB2*. However, unlike Bfa1 and Bub2, Kin4 is not required for the metaphase arrest induced upon microtubule depolymerization [82,83].

Kin4 phosphorylates Bfa1 when the anaphase spindle is misplaced in the mother or when there are defective cytoplasmic microtubules (cMTs). By phosphorylating Bfa1, Kin4 inhibits Bfa1 phosphorylation by Cdc5 *in vivo *and hence promotes Bfa1-Bub2 GAP activity [82-84]. Interestingly, Bfa1 phosphorylated by Kin4 can still be phosphorylated by Cdc5 *in vitro *[84]. How Kin4 inhibits Cdc5 phosphorylation of Bfa1 *in vivo *could only be understood by protein localization studies which will be discussed in next sections (See: SPOC activation breaks the asymmetric protein localization).

**Figure 3:**
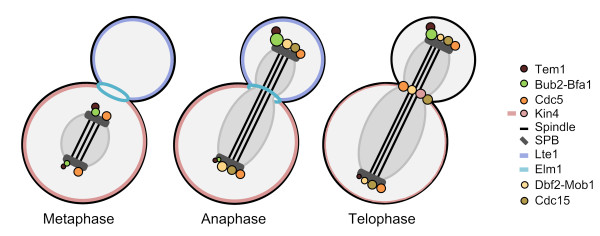
**Localization of SPOC and MEN proteins**. Localization of indicated SPOC and MEN proteins are shown in different phases of mitosis; metaphase, anaphase and telophase. Bfa1-Bub2 and Tem1 mainly localize at the dSPB (see the text for details) [10,87,88]. Cdc15 is recruited to both SPBs during late anaphase and relocates to the bud neck in late telophase [91,92,96,160]. Dbf2-Mob1 localizes on both SPBs during anaphase and accumulates at the bud neck in telophase concomitant with a decrease in the SPB localization [44,91,93]. Dbf2-Mob1 was also shown to be localized in the nucleus at late anaphase [45,46]. Cdc5 localizes to both SPBs and translocates to the bud neck in telophase [61,90,94,95]. Lte1 localizes to the bud-cortex and -cytoplasm in S, G1 and M phases. However, in telophase, shortly before cytokinesis Lte1 dissociates from the bud cortex, diffusing into the cytoplasm of the daughter and mother cells equally [10,88]. Kin4 localizes to the mother cell cortex throughout the cell cycle and to the mSPB in anaphase for a short time, and accumulates at the bud neck in telophase [53,82,83,106]. Elm1 localizes to the bud neck in mitosis but dissociates from there during telophase [111,117]. PP2A regulatory subunit Rts1 and Cdc14 are not depicted in the figure for simplicity. Rts1 localizes in the nucleus, bud neck and kinetochores [161]. Cdc14 is sequestered in the nucleolus, and released into the nucleus and cytoplasm in anaphase [19,20]. Cdc14 also associates with the SPBs in early anaphase and with the bud neck in late anaphase [25,58,89,162].

Taken together, Bfa1-Bub2, Cdc5, and Kin4 constitute the SPOC. Activity of the GAP complex is regulated by two opposing kinases; Cdc5 and Kin4. If the spindle is correctly aligned, Cdc5 phosphorylates Bfa1 and inactivates the Bfa1-Bub2 GAP complex which leads to mitotic exit. However if the spindle is misaligned or the proper microtubule cortex interactions are interfered, Kin4 kinase activates the GAP complex by phosphorylating Bfa1 and preventing the inhibitory phosphorylation of Bfa1 by Cdc5. Eventually, GAP complex delays mitotic exit through inhibition of Tem1 until spindle re-aligns in the mother-bud direction (Figure 3).

### Protein localization in an unperturbed mitosis

Experiments based on fluorescence microscopy and immuno-electron microscopy have shown that most of the MEN and SPOC components associates with the cytoplasmic surface of the SPBs and many of them translocates to the bud neck in telophase to regulate cytokinesis (Figure 3) [10,37,38,44,61,85-96]. Despite the transient SPB association of Kin4 during anaphase, localization of Kin4 and Lte1 differs remarkably from the others as they localize to the mother and daughter cell cortexes, respectively [10,82,83,87,88]. SPB localization of MEN proteins appears to be important for mitotic exit as their delocalization disturbs MEN [97]. Likewise, localization of SPOC proteins is essential for checkpoint function [3,53,98,99]. Localization of proteins implemented in SPOC and its functional importance will be described in this section. However, details on localization of other MEN proteins can also be found in Figure 3.

Bfa1, Bub2 and Tem1 localize to the cytoplasmic face (outer plaque) of the SPBs, preferentially to the bud-ward directed SPB (dSPB, daughter-directed SPB) [10]. The term "asymmetric" is widely used to describe their localization pattern in an unperturbed mitosis because from metaphase onwards they are mainly concentrated at the dSPB rather than the mSPB (mother-directed SPB) [10,88]. Bfa1-Bub2 binds to the SPB outer plaque via the SPB component Nud1, which links the γ-tubulin binder Spc72 to the central SPB protein Cnm67 [97,100-102]. SPB localization of Bfa1 and Bub2 is dependent on each other but not on Tem1. However, Tem1 association with the SPBs relies on Bfa1-Bub2 during most of the cell cycle with the exception of late anaphase [10,99]. In the absence of Bfa1-Bub2, Tem1 can bind to the SPBs only in late anaphase [10,99]. This pool of Tem1 binds equally to both SPBs and more stably than in the presence of Bfa1-Bub2 as shown by the FRAP data, suggesting that at least two different docking sites (Bfa1-Bub2-dependent and -independent) exist for Tem1 SPB association [99].

SPB-bound Bfa1-Bub2 protein amounts increase in anaphase at the dSPB with a parallel decrease at the mSPB from which Bfa1-Bub2 eventually disappears [99]. Tem1 localization resembles the localization of Bfa1-Bub2. However, Tem1 never disappears from the mSPB [10,87,88]. At the end of mitosis Bfa1-Bub2 and Tem1 amounts decrease at the dSPB concomitantly increasing at the mSPB [99] (Figure 3). The physiological importance of asymmetrically localized Bfa1-Bub2 GAP complex and Tem1 is still not clear. Disappearance of Bfa1-Bub2 from the mSPB, however, might be important for a timely mitotic exit [52,103].

How Bfa1-Bub2 and Tem1 asymmetry is established, is a question that still remains to be answered. So far, it has been shown that neither the forces generated during spindle elongation nor SPB inheritance nor passing from the bud neck affect Bfa1 asymmetry [104,105]. Actin cytoskeleton is required to initiate Bfa1 asymmetry but it is not necessary for maintenance of the already established asymmetry [105]. This is most probably due to actin function in cell polarity because some cell polarity determinants (Cdc42 and Bni1) also contribute to Bfa1 asymmetry [105]. Alternatively, actin cytoskeleton might promote Bfa1 asymmetry by facilitating correct spindle orientation.

Kin4 localization is quite different than that of Bfa1-Bub2 and Tem1. During most of an unperturbed cell cycle Kin4 associates with the cortex of the mother cell body and accumulates at the bud neck in late anaphase. However, for a short time period during mid-anaphase, Kin4 also localizes to the mSPB [82,83] (Figure 3). Accumulation of Kin4 at the bud neck is accompanied with a slight decrease in Kin4's mother specific localization. Kin4 stays at the bud neck during cytokinesis and afterwards translocates to the new bud site, where it appears only transiently [53,106] (unpublished observation of Caydasi AK).

How Kin4 associates with the cortex and SPBs, what restricts Kin4 to the mother cell or what excludes it from the daughter cell is still unclear. However, data about Kin4 localization have been accumulating over the years. It has been shown that Kin4 binds to the SPBs via Spc72 which is the SPB outer plaque component that binds γ-tubulin [84]. In addition, Clb4 and to some extend Kar9 contribute to the exclusion of Kin4 from the daughter SPB during an unperturbed anaphase. It has also been shown that the C-terminal region of Kin4 is important for its localization to the mother cell cortex and SPB [53]. Cortex localization of Kin4 might be mediated by its interaction with ergosterol [107]. Importantly, SPB and mother-cortex localization of Kin4 require Rts1, B-type regulatory subunit of the protein phosphatase 2A (PP2A) (See: Role of Kin4 localization in SPOC) [98,106].

Cdc5 localizes to both SPBs in mitosis and to the bud neck during telophase [61,90]. C-terminal polo box domain of Cdc5 is essential for both SPB and bud neck localization of Cdc5 [90]. SPB association of Cdc5 is important for MEN activation and requires the SPB outer plaque components Cnm67 and Nud1 [95]. Cdc5 phosphorylates not only Bfa1 but also Spc72 and Nud1 [84,108,109]. These phosphorylation events most likely take place at the SPBs *in vivo *because Cdc5 dependent phosphorylation of Bfa1, Nud1 and Spc72 is lost in the *nud1-2 *temperature sensitive mutant in which Bfa1, Nud1 and Spc72 are mislocalized at 37°C [84,97].

### SPOC activation breaks the asymmetric protein localization

**Figure 4:**
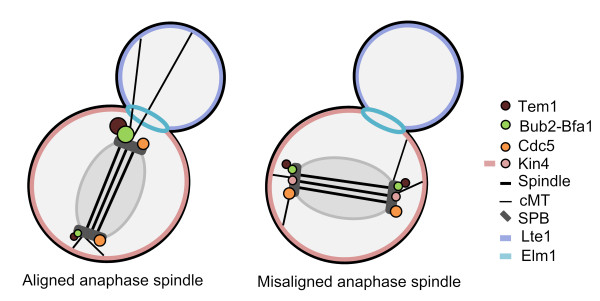
**Localization of SPOC proteins upon normal and misalignment of the anaphase spindle**. Localization of SPOC components is illustrated in anaphase when the spindles are correctly aligned, along with the mother-daughter axis (left panel) and when the spindles are misaligned (right panel). Bfa1-Bub2, Tem1 and Kin4 localization turns to symmetric from asymmetric upon spindle misalignment while Cdc5, Lte1 and Elm1 localization is not affected. See the text for details.

Upon spindle misalignment or microtubule defects (i.e. depolymerization by nocodazole) Bfa1-Bub2 and Tem1 localization changes from asymmetric to symmetric [10] (Figure 4). The term "symmetric", describes localization on both SPBs nearly equally. Similar to Bfa1-Bub2 and Tem1, SPOC activation also results in symmetric localization of Kin4 at the SPBs [82,83]. In contrast, SPB localization of Cdc5 does not change in response to spindle misalignment [84].

Despite the lack of mechanistic understanding for the establishment of Bfa1-Bub2 and Tem1 asymmetry, more insight has been gained into how the asymmetry is broken. The order of the events most likely starts with the change in Kin4 localization which is triggered by SPOC activating conditions, like misaligned spindles or defective microtubules (see the section: Sensory mechanisms for SPOC activation). Thereby, Kin4 gets access to Bfa1 and phosphorylates it on at least two residues (S150 and S180) [84].

What is the functional consequence of Bfa1 phosphorylation by Kin4? Careful FRAP measurements have shown that Bfa1-Bub2 is stably associated with the dSPB during an unperturbed cell cycle (t_1/2 _> 200 s). However, phosphorylation of Bfa1 by Kin4 loosens Bfa1-Bub2 interaction with the dSPB and promotes rapid exchange (t_1/2 _≈ 20 s) of the Bfa1-Bub2 GAP complex at both SPBs [99,105]. This causes a decrease in SPB bound Bfa1-Bub2 amounts accompanied by an increase in the cytoplasmic pool of the GAP complex [99]. Cells, in which Bfa1-Bub2 is constitutively targeted on both SPBs symmetrically but "stably", are SPOC deficient; indicating that the change in Bfa1-Bub2 SPB binding dynamics is essential for SPOC activity [99].

How is Bfa1-Bub2 GAP complex kept so efficiently active during SPOC? Dissociation of Bfa1-Bub2 from the SPBs is most likely a way of keeping Bfa1 away from the inactivating action of the polo like kinase Cdc5, which phosphorylates Bfa1 at the SPBs [84,99]. So far, it is unclear whether there is a phosphatase responsible for removing the phosphates from Cdc5 phosphorylated sites in Bfa1. Nevertheless, the rapid turnover of Bfa1 phosphorylated by Kin4 at SPBs might be sufficient to explain how Kin4 counteracts Cdc5 so efficiently *in vivo *but not in the *in vitro *system that lacks SPBs and any kind of compartmentalization [84,99].

On the other hand, Tem1 association with the SPBs is highly dynamic in the presence of Bfa1-Bub2 (t_1/2 _≈ 3 s) regardless of the cell cycle stage and the spindle alignment status [87,99]. Tem1 amounts also decrease on the SPBs of misaligned spindles [87,99]. Given that Tem1 association with the SPBs is via Bfa1-Bub2 except during late anaphase, the decrease in Tem1 SPB localization upon spindle misalignment likely follows the decrease in Bfa1-Bub2-SPB binding [10,99]. Consequently, during spindle misalignment both the Bfa1-Bub2 GAP complex and the GTPase Tem1 is mostly found dispersed in the cytoplasm rather than at the SPBs. The mechanism by which cytoplasmic Bfa1 and Bub2 inhibit Tem1 is not clear. It is tempting to speculate that GAP activity of the cytoplasmic complex inhibits the GTPase mainly in the cytoplasm. The fact that Bfa1-Bub2 is able to promote GTP hydrolysis of Tem1 *in vitro *in the absence of SPBs further supports this notion. Rigorous biochemical analysis will be however necessary to clarify the molecular mechanism of Tem1 inhibition by Bfa1-Bub2 upon SPOC activation.

### Role of Kin4 localization in SPOC

Role of Kin4 in SPOC function involves regulation of both its localization and its activity. SPB localization of Kin4 is essential for its role in activating Bfa1-Bub2, as preventing Kin4 SPB binding by targeting Kin4 constitutively to the cell cortex results in SPOC deficiency. In addition, constitutive targeting of Kin4 to the SPB outer plaque (via a Kin4-Spc72 chimera) is able to cause a 10 min delay in mitotic exit by promoting some Bfa1-Bub2 turnover (t_1/2 _≈ 40 s) at the SPBs even when the anaphase spindle is correctly aligned [84,99]. Yet, constitutive targeting of Kin4 to the SPBs is not adequate for SPOC proficiency, indicating that disruption of either SPB or cortex binding of Kin4 impairs proper Kin4 function when the spindle is misaligned [53,98].

PP2A B-type regulatory subunit Rts1 is required for Kin4 SPB and cortex binding [98,106]. Rts1 promotes dephosphorylation of Kin4, either directly or indirectly, and probably establishes Kin4 localization through this dephosphorylation. Besides, *rts1Δ *cells fail to arrest in response to spindle misalignment although Kin4 kinase activity is not affected, emphasizing the significance of Kin4 localization for checkpoint integrity [98,106].

Chan and Amon (2010) recently demonstrated the importance of Kin4's C-terminal region for Kin4 localization and SPOC activity. Overexpression of the Kin4 kinase domain, which resides at the N-terminal region of Kin4, is enough to inhibit mitotic exit but its expression at endogenous levels is not sufficient to keep the anaphase arrest upon spindle misalignment. Furthermore, Kin4 lacking the C terminal 146 amino acids cannot localize to the mother cell cortex and the SPBs and thus it is unable to engage SPOC. Finally, a single amino acid substitution (F793A) at the C terminal region prevents cortical localization of Kin4 (as well as reducing the SPB localization) and results in SPOC deficiency [53]. All aforementioned data indicate that Kin4 localization relies on the C terminal region of Kin4 and SPOC proficiency is tightly coupled to proper Kin4 localization. Interestingly, mutation of a serine residing in the C-terminus of Kin4 to an alanine (S508A) results in Kin4 mislocalization on both mother and daughter cortexes suggesting that Kin4 C-terminal is also important for restriction of Kin4 to the mother cell cortex [53].

### Elm1 regulation of Kin4 catalytic activity

Kin4 kinase activity is absolutely vital for SPOC function. Lately, we and others have shown that Kin4 kinase activity requires another kinase, namely Elm1 [106,110]. Elm1 is a bud neck localized kinase which is responsible for regulation of many other kinases including Hsl1, Gin4, Snf1 and Cla4 [111-114]. Together with Hsl1, Gin4 and Kcc4, Elm1 is one of four bud neck kinases controlling proper septin ring assembly, cytokinesis and the morphogenesis checkpoint (a checkpoint that delays entry into mitosis in response to polarization defects) [111-114]. Deletion of *ELM1*, or replacement of the wild type *ELM1 *with a kinase dead allele rescues the toxicity of *KIN4 *overexpression. In addition, *elm1 *cells are deficient in keeping the SPOC arrest in response to spindle misalignment [106,110]. This is because Kin4 is catalytically inactive in cells lacking *ELM1 *[106]. Elm1 is directly responsible for phosphorylation of a threonine (T209) residue within the Kin4 kinase activation loop (T-loop) which is essential for full activation of Kin4 [106].

Several protein kinases are regulated through T-loop phosphorylation. A dephosphorylated T-loop acts as an autoinhibitor by blocking substrate access to the active site or by blocking ATP binding [115]. Hence, T-loop phosphorylation is an excellent way of regulating kinase activity. Unexpectedly, Kin4 T-loop phosphorylation does not increase in response to SPOC activation, neither does Kin4 kinase activity [106] (unpublished observation of Caydasi AK). In this regard, it is important to keep in mind that Kin4 activity is also regulated by its localization *in vivo*. It is, therefore reasonable to hold Kin4 in an active state and change its localization as a response to spindle misalignment. Consequently, it is unlikely that Elm1 is either a sensor or a protein related with the sensor of the SPOC, but Elm1 phosphorylation of Kin4 at T209 residue is indispensable for Kin4 kinase activity and SPOC function [83,84,106,110].

Kin4 T-loop phosphorylation by Elm1 persists throughout the cell cycle, with an increase in mitosis [106]. Kin4 kinase activity exhibits a similar pattern [83]. So far, no phosphatase is known to be removing the phosphate at the T209 residue. Thus, fluctuation in T209 phosphorylation might be a reflection of Elm1 protein levels that increase in mitosis and decrease at the end of mitosis likely by degradation through a mechanism dependent on its phosphorylation by Cdk [111,114,116]. At present, the physiological importance of down regulation of T209 phosphorylation at the end of mitosis is unclear. Kin4 mutants that permanently mimic the phosphorylation at T209 residue (Kin4-T209D) do not exhibit prolonged mitosis [110]. This suggests that reduction in T209 phosphorylation is not essential for timely exit from an unperturbed mitosis. One possibility is that, the decrease in ratio of T-loop phosphorylated Kin4 at the end of mitosis could be important for down regulation of Kin4 activity upon re-alignment of a previously misaligned spindle, and so it might be important for mitotic exit. Alternatively, basal levels of Kin4 activity might be important for phosphorylation of yet unknown targets of Kin4.

Elm1 localizes to the bud neck as soon as a bud neck forms and dissociates from there prior to cytokinesis. Elm1 bud neck localization depends on the septin Cdc12 and is mutually required for proper localization of the septins Cdc12 and Cdc11 [111,117]. Bud neck localization of Elm1 has been shown to be important for Elm1 function in regulating the morphogenesis checkpoint but it is not required for activating the kinase Snf1 (a kinase involved in metabolic regulation under stress conditions, mainly during glucose starvation) [118]. The role of Elm1 bud neck localization is less clear in SPOC. Delocalized Elm1 can still activate Kin4 kinase via T209 phosphorylation [106]. However, cells carrying a C-terminally deleted *ELM1 *allele which cannot localize to the bud neck were reported to be SPOC deficient [110]. It is thus likely that Elm1 bud neck localization contributes to SPOC function via a mechanism different than T-loop phosphorylation.

In addition to T209 phosphorylation, Elm1 phosphorylates other residues in the C-terminal region of Kin4 *in vitro*. Mutation of these phosphorylation sites to alanine results in mild SPOC deficiency without affecting Kin4 kinase activity and localization [106]. At present, the reason behind this SPOC deficiency is unclear. It could be via subtle modulations of Kin4 SPB and cortex binding dynamics which were not resolved by the still image analysis. Alternatively, it could be via alteration of Kin4 binding to the bud neck, although the significance of Kin4 bud neck localization is not yet clear. Indeed, in *elm1Δ *cells, Kin4 localizes to the SPBs as in wild type cells and to the mother cell cortex only with slightly reduced efficiency, whereas bud neck localization of Kin4 is significantly reduced [106,110].

Kin4 kinase activity appears to be dispensable for Kin4 SPB and cortex localization because a kinase dead mutant of Kin4 and Kin4 of *elm1Δ *cells can still localize on both sub-cellular positions [84,106]. Hence, Kin4, which is not activated by Elm1, can still be targeted to the SPBs and to the cell cortex possibly in an Rts1 dependent manner. However, the appearance of hyperphosphorylated forms of Kin4 in *rts1Δ *cells requires Elm1, which supports another notion that Rts1 can act downstream and/or parallel of Elm1 in Kin4 regulation [106].

### Current Model of SPOC Activation

**Figure 5:**
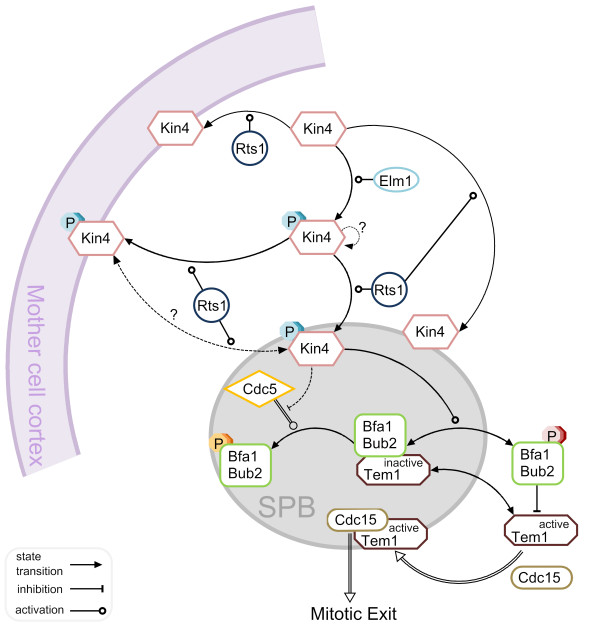
**Current model of SPOC activation**. A simple molecular network of our current understanding of SPOC function. Details of the model can be found in the text. To which structural SPB component the proteins bind is not depicted in the figure and will be explained here: Bfa1-Bub2 associates with the SPBs through Nud1 [97]. Tem1 binding to the SPBs is via Bfa1-Bub2 except for during late anaphase [10]. Kin4 binds to Spc72 [84]. Cdc5 SPB localization largely depends on Cnm67 and Nud1 although it also interacts with Spc72 [95,108]. How Cdc15 localizes to the SPBs is not clear; however it depends on Tem1 and Cnm67 and to some extend Nud1 [91,96,97]. For simplicity only one SPB (outer plaque) has been depicted and all reactions are shown in one direction except for the highly dynamic SPB binding of Tem1 and Bfa1-Bub2 which is indicated by the two-sided arrows. However, Bfa1-Bub2 independent SPB binding of Tem1 in late anaphase was not depicted as a two-sided arrow for being a relatively more stable binding than the Bfa1-Bub2 dependent pool. Dashed-lines represent the potential reactions which are not known at the moment. Double-lines refer to the reactions that favor MEN activation. Hypothetical autophosphorylation of Kin4 is indicated by a question mark.

Figure 5 represents an overview of the current model of SPOC activation. In every cell cycle, Kin4 is activated by Elm1 mainly during mitosis regardless of the spindle alignment status. In addition, Rts1 mediates the cortex and mSPB binding of Kin4. Once the spindle is misaligned, Kin4 kinase localizes on both SPBs likely in an Rts1 dependent manner and thereby phosphorylates Bfa1 [82,83,119].

Phosphorylation of Bfa1 by Kin4 increases the turn-over rate of Bfa1-Bub2 at the SPBs accompanied by a decrease in the levels of SPB associated Bfa1-Bub2. Kin4 phosphorylated Bfa1 is protected from the GAP inhibition action of the polo-like kinase Cdc5, simply because Bfa1-Bub2 dissociates from the SPBs and therefore it is kept away from Cdc5 which phosphorylates Bfa1 at the SPBs [84,99].

Together with Bfa1-Bub2; Tem1 is also released into the cytoplasm because Tem1 binds to the SPBs mainly via Bfa1-Bub2 [10,99]. Hence, the GAP complex is released into the cytoplasm, probably where it inhibits the Tem1 GTPase. Given that a pool of Tem1 (likely the active form) associates with the SPBs independently of Bfa1-Bub2 in late anaphase [10,99] and mitotic exit occurs without any delay in *BUB2 *and/or *BFA1 *deleted cells [10,73], we can assume that it is the Bfa1-Bub2 independent pool of Tem1 that recruits Cdc15 to the SPBs in late anaphase when the spindle is correctly aligned, triggering downstream events in MEN. Thus, it is tempting to speculate that upon SPOC activation Tem1 which is kept away from the SPB and inhibited in the cytoplasm cannot bind to the Bfa1-Bub2-independent docking site at the SPBs where it is supposed to meet with its target, Cdc15. Hence, mitotic exit is inhibited until spindle misalignment is corrected. When the spindle re-aligns in the mother-bud direction, this process is most likely reversed because the mitotic exit activator Lte1 resides in the daughter cell compartment and Kin4 is excluded from there.

### Sensory mechanisms for SPOC activation

SPOC gets activated when the mitotic spindle misaligns in the mother cell. This could be due to defects in cMT nucleation or in spindle positioning pathways. Observations indicate that loss of cMT-daughter cell cortex interactions is the main activator of Bfa1 symmetry rather than loss of mitotic spindle integrity or cMT-mother cortex interactions [105]. Treatment of the cells with nocodazole like drugs also activates SPOC, mainly because they depolymerize the microtubules diminishing cMT-cortex interactions. It is not known how the information is transferred from the cMT-cortex interaction to Kin4, but apparently Kin4 localization on both SPBs is triggered in response to loss of contact between cMTs and bud cell cortex.

It is also worth mentioning that, cMT-bud neck contact was also reported to be important in SPOC arrest in a way that persistent loss of cMT-bud neck interaction causes SPOC failure [120,121]. Therefore, SPOC might monitor the presence of cMTs in the bud neck. However, this has likely a small contribution to SPOC activation, if at all, because of the low penetrance of the phenotype [120,121]. It may still be possible that the presence of cMTs at the bud neck and loss of cMT-daughter cell cortex interactions might additively promote SPOC arrest.

Moreover, SPB itself might be a part of the SPOC sensory mechanism. Kin4 binds to the SPBs via the γ-tubulin receptor Spc72 [84]. Nevertheless, Spc72-7 mutants that are able to recruit Kin4 to the SPBs are still SPOC deficient, indicating that Spc72 might have a function in SPOC other than providing a docking site for Kin4 [84].

What is the molecular mechanism sensing cMT-cortex interaction? We could learn from the sensory machinery of other mitotic checkpoints like spindle assembly checkpoint (SAC). SAC senses the occupancy of the kinetochores by the microtubules and the lack of tension between the sister kinetochores [122]. It has been well established that the kinetochores which are not yet attached to the spindle microtubules recruit the SAC components keeping the SAC active. Whereas, microtubules attached to the kinetochores promote the removal of these proteins, inhibiting the SAC machinery (i.e. Mad1-Mad2 complex) [123-125]. On the other hand, kinetochores attached to the microtubules in a syntelic or monotelic manner are occupied but not under tension. In this case, the conserved protein kinase Aurora B (Ipl1 in budding yeast) promotes the detachment of the microtubules from the kinetochores by phosphorylating key substrates including Dam1 and Ndc80 complexes [126-132]. Aurora B, localizing to the innercentromeric region, has access to its substrates at the kinetochore only in the absence of an intrakinetochore tension, likely due to spatial separation [133-138].

It would be interesting to understand if any similarity exists between SPOC and SAC sensory mechanisms. The fact that disruption of the spindle microtubules *per se *does not activate SPOC, indicates that tension created on SPBs by the spindle forces are not involved in SPOC activation [105]. However, we cannot exclude the possibility that the tension created on the SPB outer plaque through cMTs might trigger SPOC activation. Alternatively, loss of cMT-daughter cortex interactions might be transmitted to the SPOC components by a mechanism similar to sensing of an unattached kinetochore. It is possible that some factors transferred along the cMT from the bud cortex to the dSPB might inhibit Kin4 binding to the dSPB when the cMTs are attached to the bud cortex. Likewise, absence of cMT-cortex interactions could generate a signal that modifies Kin4 allowing for its SPB binding. These are only hypothesis at the moment and more research is needed for elucidation of the true sensor for SPOC.

### SPOC like mechanisms in higher eukaryotes

Spindle orientation along the polarity axis is vital in asymmetric cell divisions to assure the outcome of the division is asymmetric. Therefore it is likely that checkpoints ensuring correct spindle positioning exist in higher eukaryotes too. Interestingly, studies from Yamashita and colleagues indicate the presence of a checkpoint, monitoring centrosome orientation in *Drosophila *male germ line stem cells [139,140]. Centrosome orientation checkpoint monitors the position of the centrosomes with respect to the position of the hub and delays entry into mitosis when centrosomes fail to align perpendicularly to the hub. The frequency of centrosome misalignment increases with the age of the fly. Therefore the number of stem cells that can undergo mitosis decreases as the organism ages. Thus, spermatogenesis declines in elderly flies without a need for reduction in the stem cell number [139,140]. Many other studies established the existence of a preferred direction of spindle orientation in asymmetric cell divisions of other cell types including basal epidermal cells, intestinal stem cells, and neuronal stem cells [139,141-146]. It would be interesting to ask whether SPOC or centrosome alignment checkpoint like mechanisms exist in those systems too.

**Figure 6:**
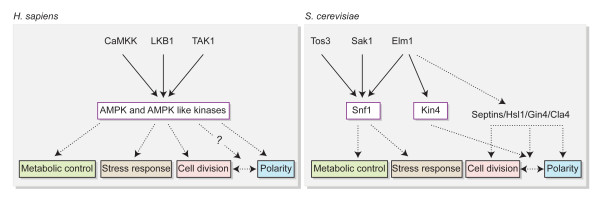
**Snf1 and AMPK pathways**. Analogy between budding yeast's Snf1 and mammalian AMPK pathways is illustrated. Elm1/Sak1/Tos3 redundantly activates Snf1 which is involved in metabolic control and stress response. LKB1/CaMKK/TAK1 activates AMPK which is implicated in metabolic control, stress response, cell division cycle and cell polarity. Kin4 is activated by Elm1 and mediates coordination of mitotic exit with spindle alignment along the polarity axis. Elm1 is also involved in regulation of other proteins related with polarity and cell division. Solid lines indicate direct activation of the protein catalytic activity. Dashed lines represent involvement of the indicated proteins in the regulation of the corresponding proteins or pathways directly or indirectly.

Functional higher eukaryotic equivalents of Tem1, Bfa1-Bub2 and Kin4 have not been identified so far. Nevertheless, homologues of the downstream MEN components Cdc15 and Dbf2-Mob1 exist in Salvador-Warts-Hippo pathway (SWH, pathway that controls organ size) of *Drosophila *and human [147]. In addition, Elm1 is known to be involved in a pathway the homologue of which exists in mammals. Elm1 together with Sak1 and Tos3 activates Snf1 (yeast homologue of mammalian AMPK, AMP-activated protein kinase) [148-150] (Figure 6). AMPK is activated by mammalian LKB1, CaMKK and TAK1 kinases. Interestingly, Elm1/Sak1/Tos3 function in regulating Snf1 in budding yeast can also be fulfilled by human LKB1/CaMKK/TAK1 kinases indicating that they are highly conserved [151]. Budding yeast's Snf1 is important for metabolic control, especially in response to stress conditions [151,152]. On the other hand, in fruit fly and mammals, AMPK is implemented in many cellular pathways involving cell cycle, cell polarity, metabolic control and stress response [153,154]. Given the analogy between the yeast Snf1 pathway and mammalian AMPK pathway (Figure 6), what fulfills the cell cycle and polarity functions in Snf1 pathway? Kin4 appears to be a good candidate as it acts by coordinating mitosis with spindle alignment along the polarity axis. Besides, Kin4 has been categorized in Snf1/AMPK family kinases belonging to the major group CaMK (Ca^2+^-calmodulin-dependent protein kinase) upon amino acid sequence similarity [155-157]. Consequently, further molecular studies of SPOC and functional identification of mammalian counterparts of yeast homologues might help to shed light onto related pathways contributing to accuracy of asymmetric cell divisions in higher eukaryotes.

## Conclusion

Every cell division is inherently asymmetric in *S. cerevisiae *and relies on correct positioning of the mitotic spindle along the polarity axis for maintenance of ploidy. This makes budding yeast an excellent model organism to study coordination of asymmetric cell division with correct spindle orientation. SPOC was discovered in budding yeast as a mechanism monitoring the spindle direction and halting cell cycle progression in response to spindle misalignment. Since then, our knowledge about SPOC is growing, but we are far from fully understanding the molecular mechanisms behind.

At the moment the main mystery is how the positional cue (likely from cMT-bud cortex) is transmitted to the SPOC activating kinase Kin4. Elm1 and Rts1 are upstream elements controlling Kin4 activity and localization. However, Elm1 activates Kin4 regardless of the spindle direction and we lack information about how exactly Rts1 contributes to Kin4 localization. Analysis of how Kin4 is localized to the cell cortex and to the SPBs might lead us to understand Rts1's role in Kin4 regulation and might help us to find other regulators of Kin4.

Another ambiguity is how the MEN gets activated, and especially how SPOC is inactivated after re-alignment of a previously misaligned spindle. Polarized localization of the SPOC activator Kin4 to the mother and MEN activator Lte1 to the daughter cell could be an explanation for this. However, what happens in reality is probably more complex than that, because Lte1 is not essential for mitotic exit at physiological temperatures and the role of Lte1 in MEN activation is so far uncertain.

Is Kin4 pathway the only way of GAP activation? Analysis of protein binding dynamics shows that Kin4 is the major factor promoting Bfa1-Bub2 turnover at the SPBs. However, even in cells lacking Kin4, Bfa1-Bub2 binding to the SPBs becomes slightly dynamic upon misorientation of the anaphase spindle. This observation suggests that yet unidentified pathways might also regulate Bfa1-Bub2 in parallel to Kin4 as a response to spindle misalignment. Indeed, Bfa1-Bub2 is also required for metaphase and G2/M arrests due to spindle or DNA damage respectively. In addition, the phosphorylation status of Bfa1 and Bub2 changes as a response to SAC and DNA damage checkpoint activation [12,77,79,158,159]. Thus, there are other means of regulating Bfa1-Bub2, but if any of those also contribute to the SPOC arrest is still not known.

In conclusion, more work has to be done to illuminate the SPOC field. Like in every concept, the more insight we gain, it is likely that the more unknowns we will face. We believe that elucidation of the mechanisms by which SPOC works will progress faster in this era of molecular and systems biology.

## Competing interests

The authors declare that they have no competing interests.

## Authors' contributions

AKC wrote the manuscript with help of BI and the critical input of GP. AKC and BI designed and prepared the figures. All authors read and approved the final manuscript.

## Authors' information

GP is the group leader of the Helmholtz junior research group Molecular Biology of Centrosomes and Cilia (MBCC) at German Cancer Research Center (DKFZ) in Heidelberg/Germany. AKC is a PhD student and BI is a postdoctoral fellow. We are interested in understanding how spindle orientation and cytokinesis are coordinated with chromosome segregation in budding yeast and how ciliogenesis occurs in mammalian cells.
